# The Influence of Aging, Hearing, and Tinnitus on the Morphology of Cortical Gray Matter, Amygdala, and Hippocampus

**DOI:** 10.3389/fnagi.2020.553461

**Published:** 2020-12-04

**Authors:** Oliver Profant, Antonín Škoch, Jaroslav Tintěra, Veronika Svobodová, Diana Kuchárová, Jana Svobodová Burianová, Josef Syka

**Affiliations:** ^1^Department of Auditory Neuroscience, Institute of Experimental Medicine of the Czech Academy of Sciences, Prague, Czechia; ^2^Department of Otorhinolaryngology, 3^rd^ Faculty of Medicine, Faculty Hospital Kralovske Vinohrady, Charles University, Prague, Czechia; ^3^MR Unit, Institute of Clinical and Experimental Medicine, Prague, Czechia; ^4^Department of Otorhinolaryngology and Head and Neck Surgery, 1^st^ Faculty of Medicine, University Hospital Motol, Charles University, Prague, Czechia

**Keywords:** presbycusis, tinnitus, auditory system, limbic system, morphometry

## Abstract

Age related hearing loss (presbycusis) is a natural process represented by elevated auditory thresholds and decreased speech intelligibility, especially in noisy conditions. Tinnitus is a phantom sound that also potentially leads to cortical changes, with its highest occurrence coinciding with the clinical onset of presbycusis. The aim of our project was to identify age, hearing loss and tinnitus related structural changes, within the auditory system and associated structures. Groups of subjects with presbycusis and tinnitus (22 subjects), with only presbycusis (24 subjects), young tinnitus patients with normal hearing (10 subjects) and young controls (17 subjects), underwent an audiological examination to characterize hearing loss and tinnitus. In addition, MRI (3T MR system, analysis in Freesurfer software) scans were used to identify changes in the cortical and subcortical structures. The following areas of the brain were analyzed: Heschl gyrus (HG), planum temporale (PT), primary visual cortex (V1), gyrus parahippocampus (PH), anterior insula (Ins), amygdala (Amg), and hippocampus (HP). A statistical analysis was performed in R framework using linear mixed-effects models with explanatory variables: age, tinnitus, laterality and hearing. In all of the cortical structures, the gray matter thickness decreased significantly with aging without having an effect on laterality (differences between the left and right hemispheres). The decrease in the gray matter thickness was faster in the HG, PT and Ins in comparison with the PH and V1. Aging did not influence the surface of the cortical areas, however there were differences between the surface size of the reported regions in the left and right hemispheres. Hearing loss caused only a borderline decrease of the cortical surface in the HG. Tinnitus was accompanied by a borderline decrease of the Ins surface and led to an increase in the volume of Amy and HP. In summary, aging is accompanied by a decrease in the cortical gray matter thickness; hearing loss only has a limited effect on the structure of the investigated cortical areas and tinnitus causes structural changes which are predominantly within the limbic system and insula, with the structure of the auditory system only being minimally affected.

## Highlights

- Tinnitus increases volume of hippocampus and amygdala.
- Hearing loss decreases volume of Heschl gyrus.
- Tinnitus has no effect on the structure of auditory cortex.
- Aging decreases cortical thickness, has no effect on the area of examined regions.

## Introduction

Age related hearing loss (presbycusis) and subjectively perceived phantom sound (tinnitus) are two of the most common hearing related pathologies. Hearing deterioration can begin as early as the age of 30, however it starts to manifest itself as high frequency hearing loss and worsened speech perception at around the age of 60 (Gates and Cooper, 1991), affecting ~30% of the population (Lin et al., 2011). The condition becomes even more pronounced with increasing age, reaching 45–67% in 7th decennium and ~75–80% in the 8th decennium (Agrawal et al., 2008). Tinnitus prevalence in the population is estimated to be around 2–3% and varies with age (Bhatt et al., 2016), reaching ~15% in the 7th decennium (Shargorodsky et al., 2010). Approximately 90% of patients with chronic tinnitus also suffer from clinically relevant sensorineural hearing loss (SNHL) (Adrian and Refaie, 2000). The remaining 10% have normal audiograms; however this does not necessarily mean normal auditory function. On the other hand, a high number of patients with SNHL never develop tinnitus.

The degree of presbycusis varies based on several factors, such as hormonal changes (Pearson et al., 1995), occupational hazards, cardiovascular diseases (Helzner et al., 2005), diabetes (Horikawa et al., 2013), lack of exercise and lower social-economic status (Emmett and Francis, 2015) and genetic susceptibility to sensorineural hearing loss (Momi et al., 2015). Similarly, tinnitus can be also caused by various pathologies, most commonly cochlear lesion as a result of chronic or acute SNHL induced by noise and/or ototoxic drug exposure (Langguth et al., 2013); male gender, higher age, presence of SNHL are the most common risk factors.

Our recent audiometric analysis of the elderly population with physiologic hearing (Profant et al., 2019), showed that apart from the peripheral damage several central pathologies were also present.

In tinnitus, the acoustic characteristics of the tinnitus percept correspond with the region of hearing loss (Weisz et al., 2006), the most common sign is a notch at the tinnitus related frequency (Norena et al., 2002). If tinnitus is present in the elderly population, it negatively influences the ability to detect tones in noise, it also seems that speech comprehension of the elderly with tinnitus is more dependent on temporal modulation and interaural time delay, suggesting slightly different auditory processing at suprathreshold levels (Bureš et al., 2019).

Although tinnitus is in principle multifactorial pathology that influences the overall mental state of the affected patient, it often has an auditory origin. Tinnitus may cause fear, stress, anxiety and depression that potentially lead to decreased concentration and cognitive dysfunction (Baguley et al., 2013). A similar pathological state may be induced by presbycusis, in which case the symptoms may comprise stress, depression and cognitive impairment (Tu and Friedman, 2018).

As previously reported, although both pathologies presumably originate in the inner ear, there is clear audiologic evidence concerning their central components.

In the human AC, functional imaging showed tonotopic map reorganization as a necessity for chronic tinnitus (for review see Eggermont, 2006), however this concept has been questioned (Langers et al., 2012) and recently Koops et al. (2020) showed that tinnitus alters the reorganization of AC caused by hearing loss. A functional reorganization of the AC was previously shown in presbycusis with an increased activation of the right AC in elderly subjects (Profant et al., 2015). There is limited evidence available to support morphometric changes induced by presbycusis. In our previous work (Profant et al., 2014) we showed changes in the cortical thickness and overall volume within the AC, however these changes were induced by aging itself and not by sensorineaural hearing loss. In the case of tinnitus, the area of interest has widened due to the involvement of non-auditory structures and networks, specifically the limbic system (Carpenter-Thompson et al., 2014). The involvement of the limbic system in the tinnitus network was recently supported by the direct connection between the limbic and auditory system (Chen et al., 2017). Another factor affecting the structural changes is the coincidence of hearing loss and tinnitus. Although changes of the gray matter were found in the auditory, hippocampal and thalamic areas, they were related to hearing loss rather than to tinnitus (Vanneste et al., 2015). Insular involvement in tinnitus related changes (Lenhardt et al., 2008; Vanneste and De Ridder, 2012; Shore et al., 2016), and also in sensorineural hearing loss (Xu et al., 2019), were previously reported. Six previously mentioned articles and additionally articles by Landgrebe et al. (2009), Lanting et al. (2009), Boyen et al. (2013), Yoo et al. (2016), and Schmidt et al. (2018) were used as a background for the identification of tinnitus and hearing loss related regions for our morphometric analysis.

As mentioned before, tinnitus and sensorineural hearing loss, specifically presbycusis, commonly coincide and therefore it is difficult to directly identify hearing loss, age, and tinnitus related changes. For such characterization, the careful selection of patients is necessary to control for each variable. There are several limitations of the previous studies that were avoided in the current project for which were used: age balanced groups, coherent degree of tinnitus distress, duration and laterality of tinnitus, different degrees of hearing loss accompanying tinnitus that was balanced between the tinnitus and non-tinnitus groups, statistical correction for age, intracranial volume, and degree of presbycusis.

The aim of our study was to use MR morphometry to detect and differentiate gray matter structural changes (cortical surface, thickness, and overall volume) related to aging, presence of age-related sensorineural hearing loss and its degree (presbycusis), presence of tinnitus and its specific characteristics (duration, its localization unilateral/bilateral) within different cortical regions; the Freesurfer analytic tool was used for cortical reconstruction. MR morphometry enables the detection of changes in the cortical surface, thickness, and overall volume.

## Materials and Methods

Seventy-three participants (36 males, 37 females) were examined in this study. Based on their age, hearing thresholds and presence of tinnitus, six different groups were formed: young controls (Y) with physiological hearing thresholds (normal hearing—NH) and no tinnitus (NT) (Y-NH-NT, 17 participants, average age 24.63 years old, median 23.97); a group of aged participants (O) with normal hearing for their age group (mild degree of presbycusis) represented by a physiological age-related decline of the auditory thresholds and no tinnitus (O-NH-NT, 11 participants, average age 68.19 years old, median 70.6); a group of aged participants with hearing loss (expressed presbycusis, HL) having significant elevation of the auditory thresholds compared to O-NH-NT and no tinnitus (O-HL-NT, 13 participants, average age 73.18 years old, median 73.74); a young group with normal hearing, an analog to Y-NH-NT group but with present tinnitus (Y-NH-T, 10 participants, average age 32.2 years old, median 31.34); an analog group to O-NH-NT but with tinnitus (O-NH-T, 12 participants, average age 67.19 years old, median 68.59); and analog group to O-HL-NT but with tinnitus (O-HL-T, 10 participants, average age 65.22 years old, median 64.49). The age, hearing threshold, and presence of tinnitus specificity of each group enabled differentiation between the possible independent effects of each factor.

Audiograms of all participants showed only minimal asymmetry (up to 2 dB) and therefore data from both ears were pooled together. The audiograms were then compared with average audiograms for the specific age group according to our previously published data (Jilek et al., 2014). If the audiograms fell within twice the standard deviation (2 × SD) borderline, they were considered physiologic (Y-NH-NT, Y-NH-T, O-NH-NT, and O-NH-T), if they were outside the 2 × SD borderline the hearing pathology was considered expressed (O-HL-NT and O-HL-T).

Inclusion criteria for each group were: Y-NH-NT (healthy adults with normal hearing and no tinnitus, with age <35 years), Y-NH-T (healthy adults without hearing loss, tinnitus present, with age <40 years), O-NH-NT (healthy adults with normal hearing for their age group as specified in the previous paragraph, no tinnitus and 60–80 years of age), O-NH-T (adults with normal hearing for their age group as specified in the previous paragraph, tinnitus and 60–80 years of age), O-HL-NT (adults with hearing loss as specified in the previous paragraph, no tinnitus and 60–80 years of age), O-HL-T (adults with hearing loss as specified in the previous paragraph, tinnitus, and 60–80 years of age).

All of the examined participants declined any previous otologic surgery: vestibular lesion, chronic exposure to loud noise, severe head trauma, lesion of the facial nerve, disorder of the cervical spine or had self-reported central nervous system disorder. None of the participants were musical professionals, but several in the elderly group played musical instruments sporadically (not more than once a month). An otoscopic examination, with removal of the cerumen and confirmation of an intact tympanic membrane, was performed on all of the participants. The examination procedures were approved by the Ethics Committee of the University Hospital Motol, in Prague. All participants signed written informed consent.

### Tinnitus

The presence of tinnitus was self-reported by participants. To establish the degree of tinnitus discomfort, participants completed the Tinnitus Handicap Inventory (THI) questionnaire (Newman et al., 1996). Based on their results and according to the classification scale, participants with tinnitus were divided into five categories (McCombe et al., 2001): slight (S)−19 participants, mild (Mi)−7 participants, moderate (Mo)−2 participants, severe (Sev)−1 participant, catastrophic (C)−0 participants.

Participants also reported the duration of tinnitus (at the date of audiometric examination) and tinnitus laterality (unilateral, bilateral/intracranial). The duration of tinnitus ranged from 1.5 to 8 years, with a median of 3 years. Fourteen participants suffered from unilateral tinnitus and seventeen participants suffered from bilateral/intracranial tinnitus.

### Pure Tone Audiometry

Pure tone audiometry was measured over an extended frequency range from 125 Hz to 16 kHz (specifically, at 0.125, 0.25, 0.5, 0.71, 1, 1.6, 2, 3.15, 4, 6.3, 8, 10, 12.5, and 16 kHz). Hearing thresholds were measured separately for each ear with a resolution of 2 dB.

All acoustic stimuli were delivered separately to each ear. An acoustic signal was delivered via Sennheiser HDA 200 high-frequency audiometric headphones connected to a custom-made audiometric apparatus, based on a high-quality audio device (RME Fireface), complemented by a custom-made programmable attenuator. The apparatus provided a digital-to-analog conversion and attenuation/amplification of the measurement signals, communication between the experimenter and the examined subject, and an acquisition of the subject's responses using a comfortable interface with backlit buttons (Arturia BeatStep). The apparatus was controlled by a custom-made software package built in the Matlab environment, which provided all the necessary functions including the generation and/or playback of digital measurement signals, acquisition of the subjects' responses, and basic data evaluation. The equipment was calibrated according to ISO 389-5, ISO 389-8, ISO 8253-3, and IEC 60645-3 standards using the Brüel & Kjær 4153 Artificial Ear.

### MRI Measurements

All MR measurements were performed using a Siemens Trio 3T MR system and a 12-channel head coil. T1 3D structural image was acquired by using the magnetization prepared rapid acquisition gradient echo (MPRAGE) sequence with the parameters (TI—inversion time) TI/TR/TE = 900/2,100/2.63 ms, flip angle 10°, 1 average, phase partial Fourier 6/8, matrix 256 × 256 × 256, voxel size 0.86 × 0.86 × 0.86 mm^3^, phase oversampling 40%, without slice oversampling, bandwidth 290 Hz/pixel, echo spacing 6.3 ms, without parallel imaging, prescan normalize, eliptical filter, acquisition time 9:26. Additional T2 3D structural image using 3D turbo-spin echo with variable flip angle (SPACE) was acquired with the parameters: TR/TE = 3,200/453 ms, 1.7 averages, turbo factor 167, slice turbo factor 2, slice partial Fourier 7/8, matrix 256 × 256 × 208, voxel size 0.86 × 0.86 × 0.86 mm^3^, phase oversampling 20%, without slice oversampling, bandwidth 751 Hz/pixel, echo spacing 3.5 ms, with parallel imaging iPAT GRAPPA, acceleration factor 2, reference lines 24, weak raw filter, acquisition time 8:18 was measured. The T2 image was used for bias field correction and refinement of the pial surface position in subsequent HCP pipelines/FreeSurfer data processing. Both sequences were acquired in a sagittal orientation.

### MRI Processing

Structural images were preprocessed by “PreFreeSurferPipeline.sh” script as part of HCP pipelines framework (Glasser et al., 2013). This part involved rigid-body data alignment to standard space, gradient distortion correction and bias field correction.

Volumetric segmentation and cortical surface reconstruction was done using optimized versions of recon-all script of FreeSurfer 6.0 (details of customizations see Supplementary Material). The technical details using FreeSurfer methods are described in prior publications (Dale and Sereno, 1993; Dale et al., 1999; Fischl et al., 1999a,b; Fischl and Dale, 2000; Fischl et al., 2001, 2002, 2004; Ségonne et al., 2004; Han et al., 2006; Jovicich et al., 2006; Reuter et al., 2010, 2012).

**Figure 1:**
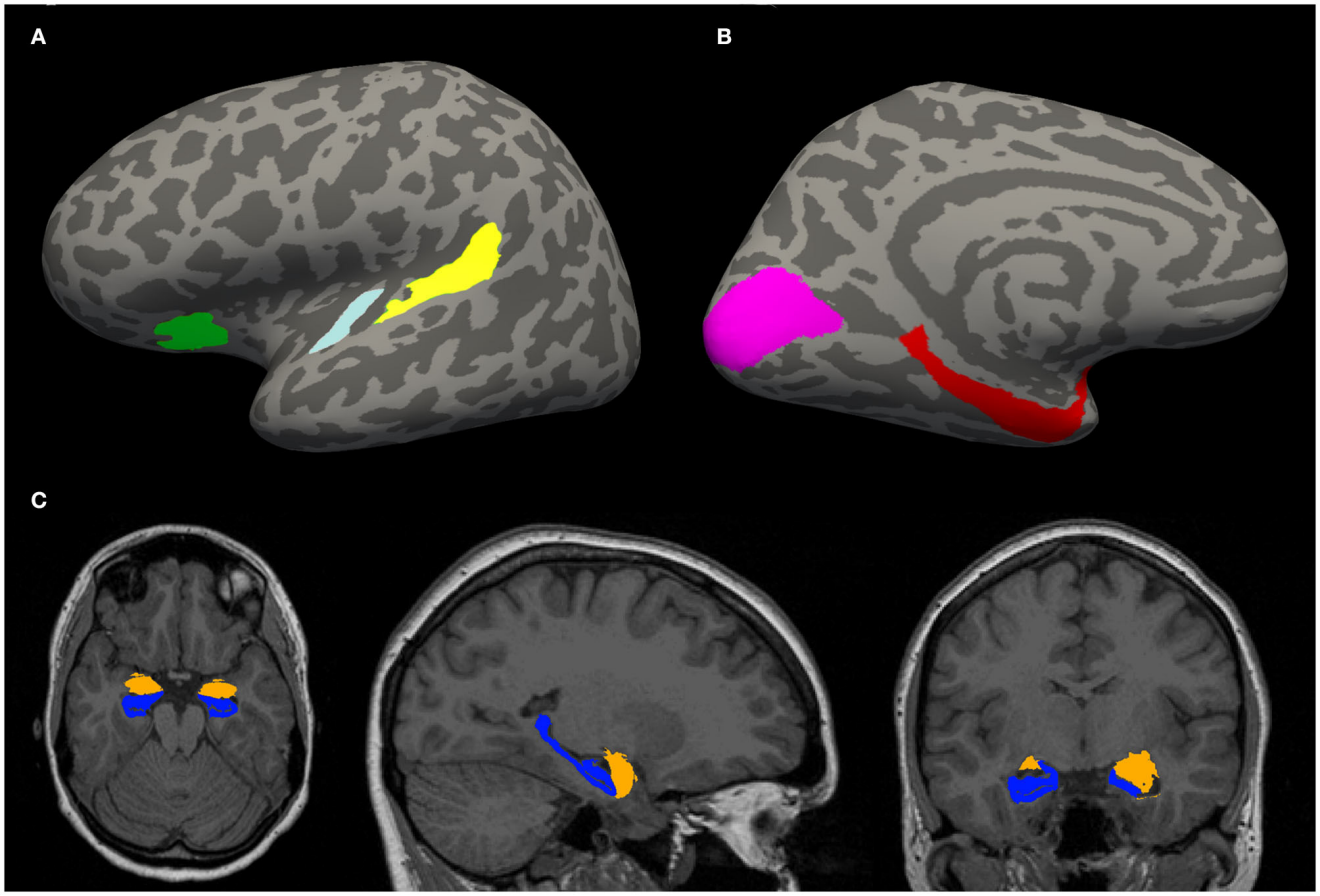
**(A,B)** Example of an inflated cortical surface with colored regions of interest. These regions were chosen based on their relation with tinnitus in the literature. Yellow—planum temporale (PT), light blue—Heschl gyrus (HG), green—anterior insula (Ins) **(A)**, purple—visual cortex (V1), red—parahippocampus (PH) **(B)**. **(C)** Example of a localization of limbic structures: hippocampus (blue) and amygdala (orange).

Morphometric parameters, such as the gyral surface area and the average thickness of the gray matter of the cortical areas of interest (Figures 1A,B) were computed.

Automatic cortical parcellation using probabilistic labeling based on gyral and sulcal structure was then performed using Destrieux probabilistic atlas (Destrieux et al., 2010). Cortical ROIs of HG, PT, PH, Ins were extracted.

For parcellation of the V1 (Brodmann area 17), the atlas provided by the Martinos Center for Biomedical Imaging and the Institute of Neurosciences and Biophysics was used (Fischl et al., 2008).

These ROIs (Figures 1A,B) were selected for analysis of morphometric parameters, such as the gyral surface area and the average thickness of the gray matter.

Additionally, whole hippocampal (Iglesias et al., 2015) and amygdalar (Saygin et al., 2017) volume (Figure 1C) was estimated by automatical segmentation module “segmentHA_T1.sh,” shipped as part of the development version of FreeSurfer.

For the hippocampus and amygdala the module provides an estimation of the volume of the whole structure (i.e., unlike cortical parcels there is no separate thickness and surface area estimation). Although there is also a subdivision to substructures available, for simplicity we only used volumes of the whole structures in our study. To increase robustness of the results, we used both T1 (using only T1 images) and T1T2 (using jointly T1 and T2 images) modes of segmentation.

Estimated intracranial volume (eTIV), which was used as a covariate in the analyses of area and volume, was derived from the determinant of the transform matrix used to align structural image with an atlas (Buckner et al., 2004).

### Region of Interest (ROI) Selection

Based on the review of the literature (Lenhardt et al., 2008; Landgrebe et al., 2009; Lanting et al., 2009; Vanneste and De Ridder, 2012; Boyen et al., 2013; Carpenter-Thompson et al., 2014; Vanneste et al., 2015; Shore et al., 2016; Yoo et al., 2016; Chen et al., 2017; Schmidt et al., 2018; Xu et al., 2019), the following regions were chosen for the ROI analysis: HG, PT, PH, Ins, V1, HP, Amg. Although not all the literature focuses on the gray matter changes, they report on the effect of tinnitus and hearing loss on the morphometry, function and connectivity within the CNS. Previously, tinnitus and hearing loss related changes were reported within the auditory pathway of humans, specifically inferior colliculus (Landgrebe et al., 2009) and also to some degree in the thalamic medial geniculate body. The auditory cortex, primary (HG) as well as secondary (PT), were reported to be affected by tinnitus (Mühlau et al., 2006; Boyen et al., 2013) and hearing loss (Husain et al., 2011; Boyen et al., 2013; Vanneste et al., 2015). Several non-auditory structures related to tinnitus and/or hearing loss were previously reported. Parahippocampus changes due to the severity of tinnitus were reported by Schmidt et al. (2018), due to the laterality of tinnitus by Vanneste et al. (2011), and due to the hearing loss by Boyen et al. (2013). Insular involvement in the tinnitus was reported by Vanneste and De Ridder (2012), and aspart of a salience network by Husain and Schmidt (2014) and Husain (2016). Limbic, specifically hippocampal and amygdalar, involvement in tinnitus related changes were reported by Landgrebe et al. (2009) and Yoo et al. (2016). All of the aforementioned regions are also affected by aging to a different degree (Fjell et al., 2014). Primary visual cortex as a sensory region, whose function is affected by aging in a similar way to the auditory cortex, was chosen as a control (as in our previous MRI reports). It should be highlighted that several other structures were reported to be involved in the tinnitus and hearing loss however these changes were either inconsistent or contradictory. Among these structures are: precuneus, cingulate cortex (several different parts were reported), cerebellum, prefrontal cortex, nucleus accumbens. Although we believe that the prefrontal cortex could play an important role in the tinnitus/hearing loss related pathology, the structure is not well-defined; respectively it is too large to be involved in the ROI morphometry and the same applies to the cingulate cortex. Therefore, to avoid the non-specificity of the whole brain approach, those structures were not involved in our analysis.

### Statistical Methods

The data of cortical thickness, surface area and hippocampal and amygdalar volume were statistically analyzed in environment R (Dalgaard, 2010) using the linear mixed-effects model as implemented in package nlme (Pinheiro and Bates, 2010).

In the models, tinnitus presence/absence (TIN), age related hearing loss (presbycusis) presence/absence (PRESB), ROI laterality (LAT), age, sex, eTIV, anatomical parcel, segmentation method (in case of hippocampus and amygdala) were modeled as fixed effects. The inter-individual variability (subject-specific offset within the dependent variable values) was modeled as a random effect. Several analyses were conducted with differing involvement of the above listed fixed effects:

M1: LAT + REGION + AGE + sex and interactions LAT:REGION + LAT:AGE + REGION:sex + REGION:AGE + LAT:REGION:AGE to compare degree of aging between selected regions and influence of laterality in all groups. The aim of M1 is to identify the effect of aging, its gradient (change over time), laterality (left vs. right hemisphere) and its interactions on the studied regions. The dependent variable is cortical thickness.

M2: for each region a separate model was fitted with effects:

TIN + PRESB + LAT + AGE + sex (+ eTIV) to test the effect of aging, age related hearing loss, tinnitus and laterality in all groups. The aim of M2 is to identify effects of aging, hearing loss, tinnitus and laterality (difference between the specific region in the left and right hemisphere) on the thickness and surface area (both were modeled as dependent variables) of each region.

M3: for each region a separate model was fitted with effects:

TIN + PRESB + LAT + AGE + sex (+ eTIV). To address the observed robust effect of aging on data from all subjects that could potentially cover non-age related changes, only data from groups O-NH-NT, O-NH-T, O-HL-NT, and O-HL-T were used for this analysis to test the effect of tinnitus and hearing. The aim of the M3 is to decrease the robust effect of aging and potentially identify effects of hearing loss and tinnitus that could be covered by impact of aging. Therefore, only aged groups were included in this model.

M4: for each region a separate model was fitted with effects:

TIN_LATERALITY + LAT + AGE + sex (+ eTIV) and interaction TIN_LATERALITY: LAT to test the effect of tinnitus laterality in tinnitus subjects from O-NH-T and O-HL-T groups. The aim of M4 is to decrease the robust effect of aging and potentially identify the effect of tinnitus laterality (unilateral vs. bilateral). Therefore, only aged tinnitus groups were included in this model (O-NH-T and O-HL-T).

M5: volume of hippocampus and amygdala was fitted with effects:

TIN + PRESB + LAT + AGE + sex + eTIV + method to test the effect of age, laterality, tinnitus, and age related hearing loss on hippocampal/amygdalar volume in all groups.

M6: in regions with a significant effect of tinnitus, the effect of tinnitus duration and severity (THI index) on a dependent variable was tested: area of planum temporale with effects TIN_LENGTH or THI + LAT + AGE + sex + eTIV in O-NH-T and O-HL-T groups. Hippocampal and amygdalar volumes were computed using T1 method with effects TIN_LENGTH or THI + LAT + AGE + sex + eTIV in Y-NH-T, O-NH-T, and O-HL-T groups.

In models with the area and volume estimated, eTIV was added as a nuisance covariate due to its effect on brain surface area. Also, sex was included as a nuisance covariate in accord with the common practice in most morphometric studies.

The statistical modeling was performed as follows: First, the Box-Cox transformation of the dependent variable to optimize normality of the model residuals was done. The search for an optimal lambda for transformation was done by iterative evaluation of the *p*-value of Shapiro-Wilk test of the residuals of a particular model. A common practice in statistical modeling is to follow the principle of parsimony and select the minimal model, i.e., retain only the terms which contribute significantly to the explanation of the variance in the data. The model simplification by using a combination of the Akaike information criterion and elimination of the non-significant terms, was therefore performed. For clarity, the structure of the full and simplified models is listed in the Supplementary Material.

The results of the final models after model simplification were further explored and interpreted using parameter estimates, scatter-plots and box-plots. Formally, each of the hypotheses was assessed by statistically testing the corresponding null hypothesis of the zero effect/difference. Two significance thresholds in the analysis were used. Firstly, the uncorrected significance level of *p* = 0.05 for each test controls the probability of false detection of each effect under the validity of the null hypothesis at 5%. However, given the multitude of hypotheses as well as the multitude of related tests for each of the hypotheses, the overall probability of spurious detection of at least one effect using the uncorrected significance threshold in the case of the validity of all null hypotheses (no effects) is far higher than 5%. Therefore, the *p*-values corrected for multiple comparisons by Holm method (Holm, 1979) were also reported.

## Results

### Assessment of the Function of the Auditory System

**Figure 2:**
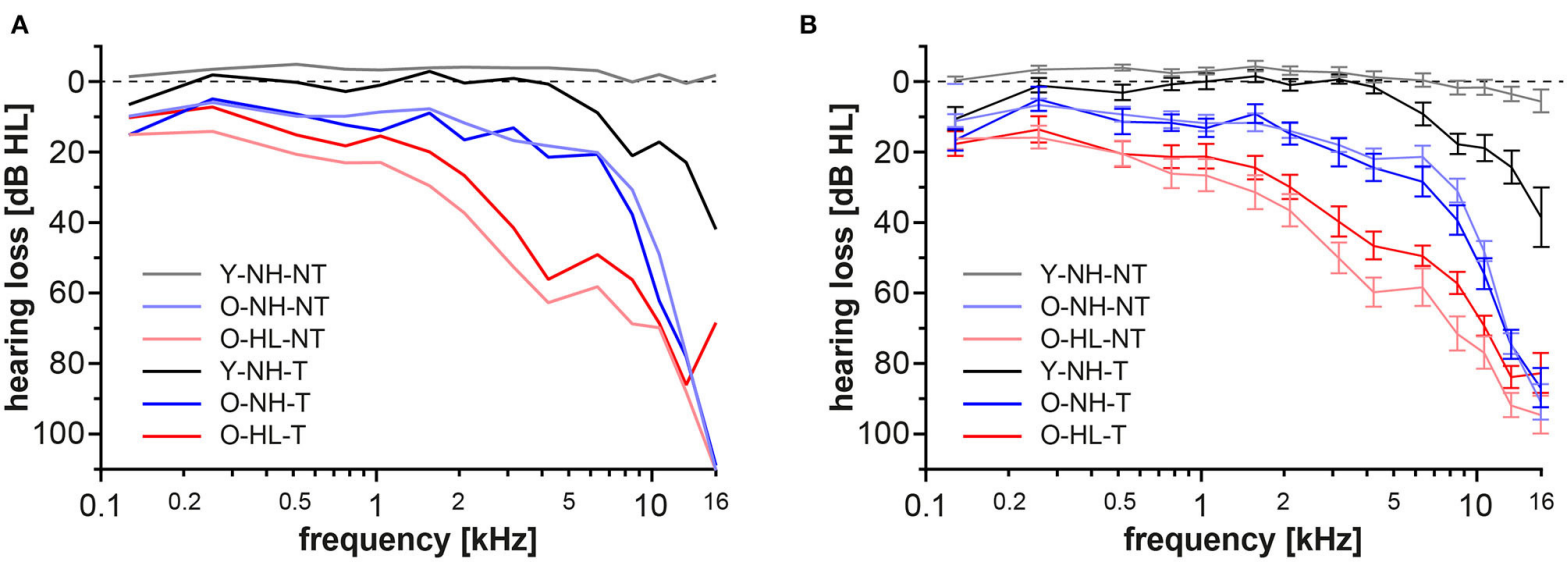
Auditory thresholds for each group in extended frequency range up to 16 kHz. Data from both ears were merged together. Values are presented as median **(A)** average± standard error of the mean (SEM) **(B)**.

The Y-NH-NT showed physiologic auditory thresholds not exceeding 20 dB HL. Patients with tinnitus and physiologic audiograms up to 8 kHz formed the Y-NH-T group, however at frequencies above 8 kHz clear elevations of the thresholds were present. In the elderly groups with normal hearing for their age group (mild presbycusis; O-NH-NT, O-NH-T) the thresholds were slightly elevated compared with both young groups, specifically at frequencies above 4 kHz, where the audiograms exceeded 20 dB HL that is considered clinically physiologic. However, these thresholds are considered normal for the age group >60 years of age, according to Jilek et al. (2014). Both groups with age related hearing loss (expressed presbycusis, O-HL-NT, and O-HL-T) showed additional deterioration of hearing with pronounced elevations of the auditory thresholds, even after the correction for age (Jilek et al., 2014) (Figure 2).

Based on our previous results on structural changes of the gray matter in patients with a different degree of presbycusis (mild vs. expressed), that showed only a minimal effect of hearing loss on thickness of the auditory cortex and examined auditory related regions (Profant et al., 2014), that were also confirmed by the results of this study, data from the O-NH-NT and O-NH-T and data from the O-HL-NT and O-HL-T were effectively pooled together to increase statistical strength.

### The Effects of Aging, Tinnitus, and Laterality on the Cortical Thickness and Surface

**Figure 3:**
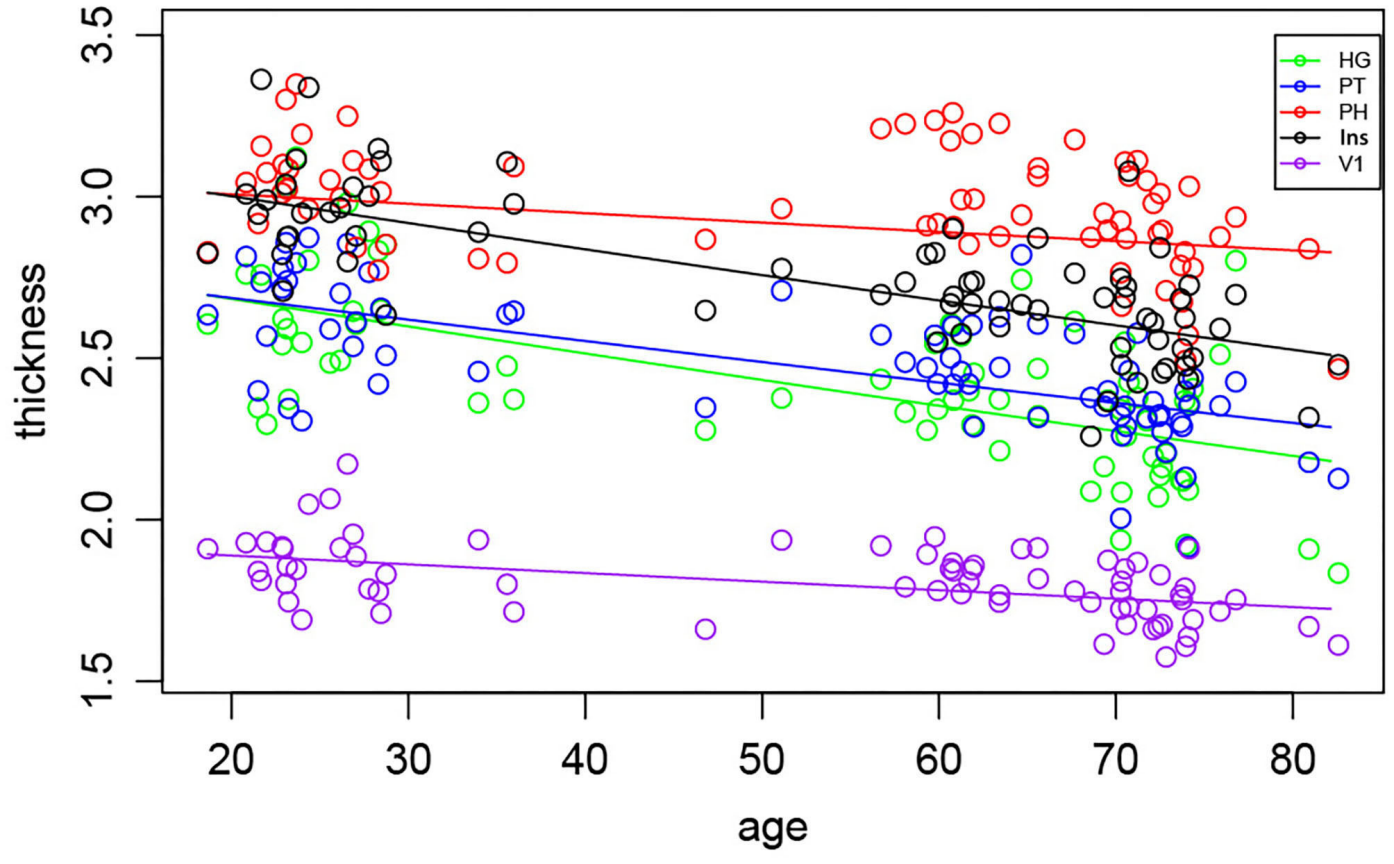
Morphometric analysis of the cortical thickness in all examined regions with the exception of the Amg and HP, shows age-related decrease. Each symbol represents one subject.

The morphometric analysis of the cortical thickness within all selected regions pooled into one statistical model (M1), showed the interaction between the age and region (Figure 3). *Post-hoc* test of the age slope PH + V1 vs. HG + PT + insula showed that PH and V1 thickness is affected less by the aging than HG, PT and insula thickness (*p* = 0.0001, corrected *p* = 0.003). No significant difference was found in the decline of the thickness between the left and right hemispheres, suggesting that the cortical gray matter thinning is symmetrical in all regions and aging had no effect on the laterality (e.g., left hemisphere declines faster than the right or vice versa).

The effects of aging, tinnitus, hearing and laterality on the cortical thickness and surface were also evaluated for each region separately (M2). The effect of age on the cortical thickness was significant in all of the regions (HG *p* < 0.0001, corrected *p* < 0.0001, PT *p* < 0.0001, corrected *p* < 0.0001, V1 *p* < 0.0001, corrected *p* = 0.0002, Ins *p* < 0.0001, corrected *p* < 0.0001, PH *p* = 0.002, corrected *p* = 0.05).

The effect of laterality on cortical thickness was present at borderline level only in the PH (*p* = 0.009, corrected *p* = 0.1) with a greater thickness in the right hemisphere.

**Figure 4:**
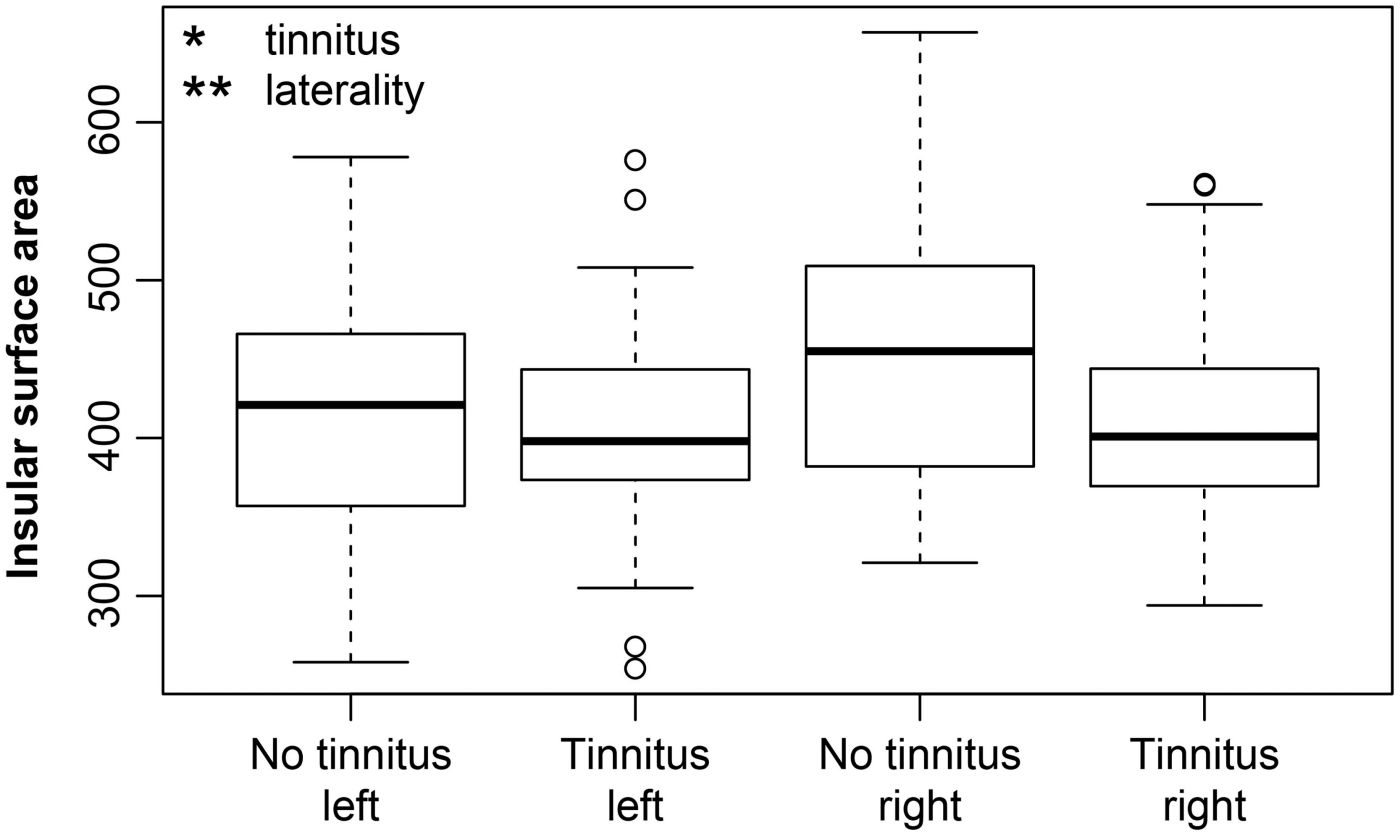
Insular surface remains stable with increasing age however, tinnitus leads to its decrease. Insular surface area is also significantly larger in the right hemisphere (laterality). The effect of tinnitus on insular surface area is independent of age or laterality. Tinnitus—include all groups with tinnitus (Y-NH-T, O-NH-T, O-HL-T), no tinnitus—include all groups without tinnitus (Y-NH-NT, O-NH-NT, O-HL-NT); right and left include data from right and left insula. *p < 0.05, **p < 0.001 uncorrected.

Regarding the extent of the surface area, the effect of aging was not observed in the examined regions with the exception of the borderline effect in the HG (*p* = 0.02, corrected *p* = 0.1). The effect of laterality was present in all of the examined regions (greater surface in the left hemisphere: HG *p* = 0.0002, corrected *p* = 0.005, PT *p* < 0.0001, corrected *p* < 0.0001, PH *p* = 0.009, corrected *p* = 0.1; greater surface in the right hemisphere: V1 *p* = 0.002, corrected *p* = 0.03, INS *p* = 0.006, corrected *p* = 0.07). Hearing loss (in our case presence of expressed presbycusis) showed only minimal effect on surface of HG (*p* = 0.04, corrected *p* = 0.14). We observed a borderline decrease of the insular surface due to the tinnitus (*p* = 0.03, corrected *p* = 0.12) (Figure 4). The surface area correlated with the intracranial volume; therefore the intracranial volume was included in the models as the nuisance covariate.

### The Effects of Hearing Loss and Tinnitus on Cortical Thickness and Surface in Elderly

Due to the strong effect of aging on our data, only data from the age coherent groups (O-NH-NT, O-NH-T vs. O-HL-NT, O-HL-T) were used to detect the possible effects of hearing loss and tinnitus (M3). In the age coherent groups, the effect of aging on cortical thickness was significant in the HG (*p* = 0.002, corrected *p* = 0.04), PT (*p* < 0.0001, corrected *p* = 0.0003), V1 (*p* < 0.0001, corrected *p* = 0.0002), PH (*p* < 0.0001, corrected *p* = 0.0001), Ins (*p* = 0.004, corrected *p* = 0.06). The significant effect of aging on cortical surface was present in the PT (*p* = 0.02, corrected *p* = 0.05) and borderline effect in PH (*p* = 0.01, corrected *p* = 0.1).

**Figure 5:**
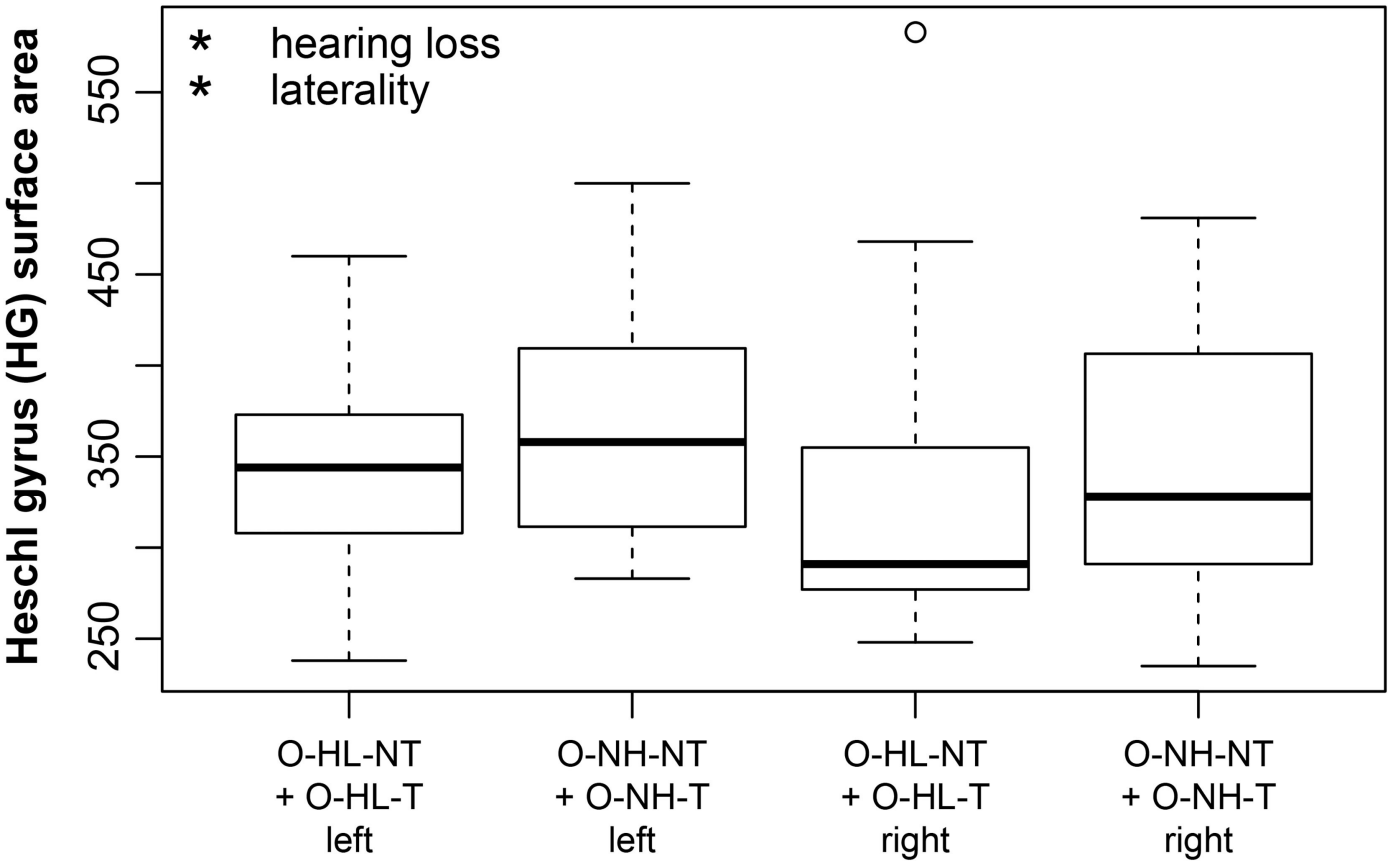
Cortical surface area is decreased due to hearing loss in HG (apparent when groups with mild and expressed presbycusis are compared). HG surface area is larger on the left side (laterality). The effect of hearing loss on surface area of HG is independent of the laterality. Only data from aged groups are used and are pooled together based on the degree of presbycusis; right and left include data from right and left HG. Data are compared based on M3 (only elderly groups). *p < 0.05 uncorrected.

**Figure 6:**
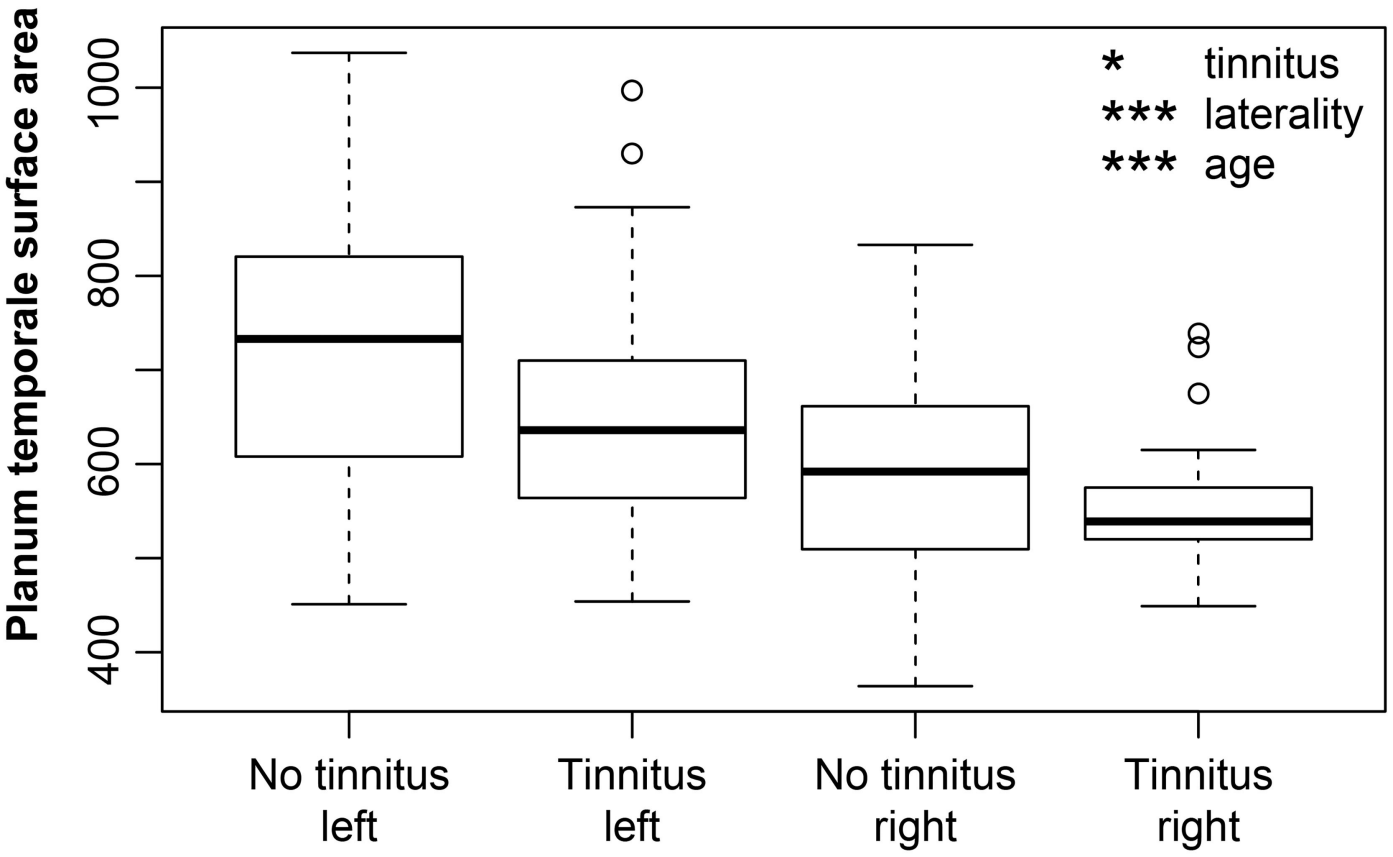
The cortical surface area is decreased due to the tinnitus in the PT (apparent when groups with mild and expressed presbycusis are compared). Left PT surface area is larger. PT surface area decreases with increasing age. Data are compared based on M3 (only elderly groups). Tinnitus—include elderly groups with tinnitus (O-NH-T, O-HL-T), no tinnitus—include elderly groups without tinnitus (O-NH-NT, O-HL-NT); right and left include data from right and left PT. *p < 0.05, ***p < 0.0001 uncorrected.

The hearing loss (comparison of the effect of mild vs. expressed presbycusis) had no significant effect in the examined areas with a borderline effect in the case of HG where the surface was found to decrease (*p* = 0.05, corrected *p* = 0.1) (Figure 5). Tinnitus had only a borderline effect on the decreased surface in the PT (*p* = 0.009, corrected *p* = 0.1) (Figure 6).

The effect of tinnitus laterality (M4) was also considered and unilateral vs. bilateral (capitis) tinnitus was examined (in groups O-NH-NT and O-HL-T). Laterality of tinnitus had no effect on the cortical thickness or surface in any of the examined regions.

### The Effects of Tinnitus and Hearing Loss on the Volume of the Amygdala and Hippocampus

**Figure 7:**
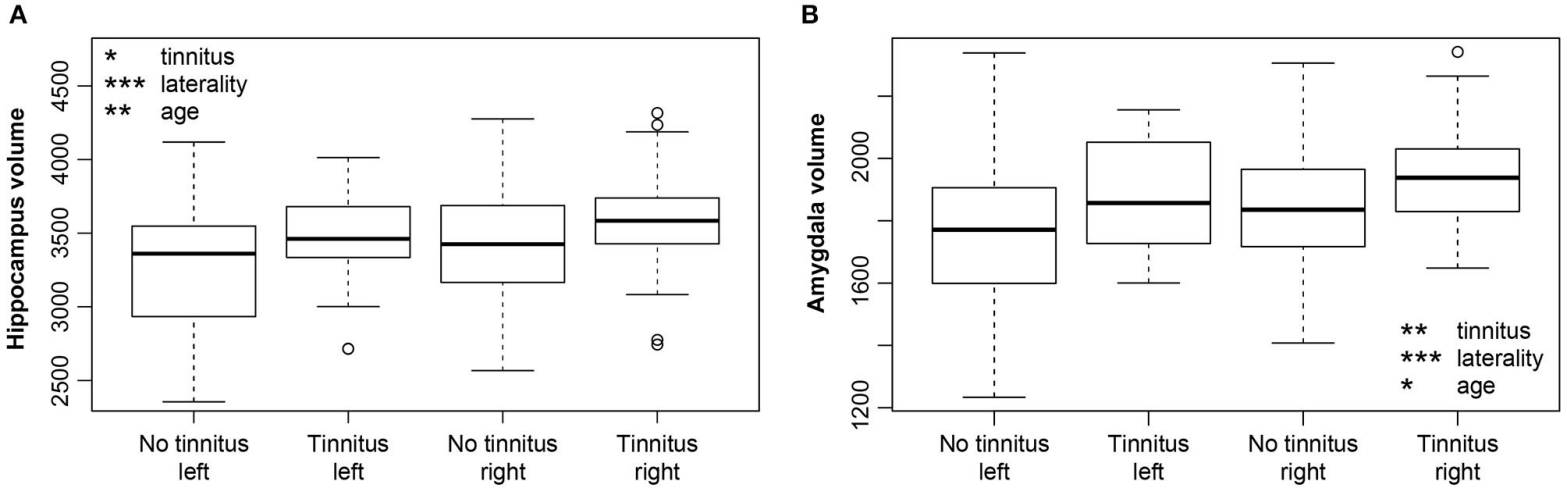
**(A)** Increase of the hippocampus volume due to tinnitus. Hippocampus is larger in the right hemisphere (laterality). **(B)** Increase of the amygdala volume due to tinnitus. Amygdala is larger in the right hemisphere (laterality). Volume of both structures decreases with age. *p < 0.05, **p < 0.001, ***p < 0.0001 uncorrected.

Hearing loss had no significant influence on the volume of either the amygdala or hippocampus (M5). However, tinnitus caused a borderline effect of the volume increase in both regions [hippocampus *p* = 0.009, corrected *p* = 0.1 (Figure 7A), amygdala *p* = 0.005, corrected *p* = 0.06 (Figure 7B)]. The duration of tinnitus and severity of the tinnitus (examined by THI) had no effect on the volume of the amygdala, hippocampus and surface of the PT (the region with an effect of tinnitus on its cortical surface) (M6). Aging had a borderline effect on both structures (HP *p* = 0.004, corrected *p* = 0.06; Amg *p* = 0.02, corrected *p* = 0.01). Laterality showed significantly greater volumes in the right amygdala (*p* < 0.0001, corrected *p* < 0.0001) as well as the right hippocampus (*p* < 0.0001, corrected *p* < 0.0001).

Statistical significance of results is summarized in Table 1.

**Table 1:** Statistical significance of examined effects shown as uncorrected *p* and corrected *p* (after correction for multiple comparisons by Holm method).

**Model (statistical analysis)**	**Examined variable**	**Region of interest**	**Examined effect**	**Uncorrected** ***p***	**Corrected** ***p***
M1	Thickness	PH+V1 vs. HG+PT+INS		0.0001	0.0029
M2	Thickness	HG	Age	0.0000	0.0000
M2	Thickness	PT	Age	0.0000	0.0000
M2	Thickness	V1	Age	0.0000	0.0002
M2	Thickness	PH	Age	0.0024	0.0456
M2	Thickness	PH	Lat	0.0086	0.1032
M2	Thickness	Ins	Age	0.0000	0.0000
M2	Area	HG	Age	0.0150	0.1032
M2	Area	HG	Lat	0.0002	0.0046
M2	Area	PT	Lat	0.0000	0.0000
M2	Area	V1	Lat	0.0015	0.0330
M2	Area	PH	Lat	0.0091	0.1032
M2	Area	Ins	Lat	0.0055	0.0715
M2	Area	Ins	Tin	0.0252	0.1035
M2	Area	HG	Presb	0.0421	0.1400
M3	Thickness	HG	Age	0.0021	0.0420
M3	Thickness	PT	Age	0.0000	0.0003
M3	Thickness	V1	Age	0.0000	0.0002
M3	Thickness	V1	Lat	0.0089	0.1032
M3	Thickness	PH	Lat	0.0350	0.1050
M3	Thickness	PH	Age	0.0000	0.0001
M3	Thickness	Ins	Age	0.0040	0.0646
M3	Area	HG	Presb	0.0467	0.1050
M3	Area	HG	Lat	0.0414	0.1050
M3	Area	PT	Tin	0.0087	0.1032
M3	Area	PT	Lat	0.0000	0.0000
M3	Area	PT	Age	0.0027	0.0486
M3	Area	V1	Lat	0.0017	0.0357
M3	Area	PH	Age	0.0141	0.1032
M3	Area	PH	Lat	0.0041	0.0646
M5	Volume	HP	Age	0.0038	0.0646
M5	Volume	HP	Lat	0.0000	0.0000
M5	Volume	HP	Tin	0.0090	0.1032
M5	Volume	Amg	Age	0.0207	0.1035
M5	Volume	Amg	Lat	0.0000	0.0000
M5	Volume	Amg	Tin	0.0045	0.0646

## Discussion

Age-related hearing loss and tinnitus are the two most common disorders affecting the auditory system. In principle both pathologies can also potentially influence non-auditory brain structures, especially if they appear at the same time. In our project we have identified several age, hearing loss and tinnitus related pathologies that alter the cortical gray matter in various regions. Age-related cortical atrophy was present in all of the examined regions, however the extent of it differed. Hearing loss and tinnitus were studied among age matched groups while controlled for the effect of age. The effect of hearing loss (degree of presbycusis) showed only a borderline effect on the surface of the AC structures, whereas the main effect of tinnitus led to an increase of the volume of structures of the limbic system (amygdala and hippocampus). The effect of aging was not significantly region-specific and did not alter the left-right hemisphere relationship.

The input criteria for the selection of volunteers were based on the goal to assemble age balanced groups: with normal hearing with and without tinnitus, with a physiologic age related decline with and without tinnitus, and elderly groups with expressed presbycusis (elevation of hearing thresholds exceeding physiologic aging) with and without tinnitus. The hearing thresholds criteria to distinguish between the mild and expressed presbycusis were based on our previous studies (Jilek et al., 2014; Profant et al., 2019). A similar approach was also used by Vanneste et al. (2015) with the exception of the higher age in our elderly groups that allowed us to test the effect of age-related hearing loss.

The aim of this study was to investigate the effects of aging, tinnitus and hearing loss on the gray matter thickness, area and volume of the auditory system and regions linked to tinnitus. In this regard, the different groups of volunteers were assembled to optimally model and test each effect. The criteria of the two elderly groups with different degrees of presbycusis (i.e., different degree of age-related hearing loss) and presence/absence of tinnitus, is what distinguishes this study from previous ones.

The most dominant finding in our study was the effect of aging on the cortical gray matter. Aging essentially, leads to multiple changes of gray matter and its effect differs among the cortical regions. Cortical atrophy caused by gray matter loss is linear in relation to aging (Allen et al., 2005). In our results, aging influences the cortical thickness in all of the examined regions. However, the size of the effect varies; the difference in the slope of thickness decreases in HG, PT and insula, compared to PH and V1. This finding is in line with the antero-posterior gradient theory (Resnick et al., 2003), according to which the frontal and parietal regions exhibit greater rates of decrease over time than the temporal and occipital regions. Although HG and PT are parts of the temporal lobe, the difference between them and V1 could be based on the much stronger effect of the cognitive impairment on the temporal regions (Sluimer et al., 2009) and could also be related to a theory that specifically middle temporal regions, which form part of the default mode network, are more strongly affected by aging (Fjell et al., 2014). Cognitive impairment is often associated with sensorineural hearing loss (Lin et al., 2013) and could potentially explain the faster cortical atrophy within the HG and PT of our elderly subjects. Apart from the strong effect on cortical thickness, aging only influences the cortical surface to a limited degree in our study, in the HG, PT, and PH. An age related decrease of the cortical surface area starts around the age of 15, but it is very mild and only becomes pronounced after the age of 45 (Schnack et al., 2015), specifically in the fronto-parietal regions that are linked to intelligence (Barbey et al., 2013), which also influences the rate of the decrease (Schnack et al., 2015). The decline of the surface area can be explained by a decrease in the dendritic neuropil (Jacobs and Scheibel, 1993) and shrinkage of the dendritic length, which is often associated with education level and leads to a decrease of the surface rather than thickness. Cortical thickness on the other hand reflects better neuroplastic alterations due to the sensory stimulation, diseases, and experience (Thompson et al., 2004; Irimia et al., 2014; Shiell et al., 2016). Therefore, as postulated by Rakic in 2007 in “radial unit hypothesis,” the surface area and gray matter thickness are not necessarily causally related to each other.

The lateralization (a difference between the regions in the left and right hemispheres) is very common in the brain regarding functional specificities, as well as the difference in volume. A typical example is the AC, where the left hemisphere is focused on the temporal parameters of sound and speech processing, whereas the right hemisphere is focused on the processing of spectral parameters and music (Zatorre and Belin, 2001; Tervaniemi and Hugdahl, 2003). As described by Profant et al. (2014), the functional disparities of the AC also have morphometric analogs: the volume and surface of the left AC is larger than the right AC and although the functional activation of the cortices balances out with increased age (Profant et al., 2015), age has no effect on the lateralization of morphometric parameters.

In principle, aging affects both the cortical surface and thickness, however the surface area is affected more consistently, whereas the thickness might even increase in some frontal and parietal regions with aging (Thambisetty et al., 2010; Chiarello et al., 2016; Dotson et al., 2016). Our results show lateralization in the cortical surface parameters, with HG, PT, and PH dominancy in the left hemisphere and V1 and Insula in the right hemisphere. PH plays an important role in cognitive processing, specifically spatial memory and navigation (Maguire et al., 1996), and is also a part of the limbic system (Li et al., 2016). Since cognition and memory are one of the first processes affected by aging, the higher age of our volunteers might affect the function as well as the morphometry of the PH. Kong et al. (2018) also reported on the effect of sex on the PH asymmetry (thickness and surface-leftward dominance). We did not test the separate effect of sex (and also other variables, such as age, hearing, and tinnitus) on the symmetry of the examined regions independently, due to the overall limited number of subjects and necessity of multiple comparison correction, which dictates the parsimonious selection of the complexity of statistical models used. The rightward asymmetry of the V1 is in agreement with our previous findings (Profant et al., 2014). From the functional perspective, the right hemisphere is more involved in the visuospatial integration processing that leads to a stronger connectivity between visual, fronto-parietal, and default mode networks within the right hemisphere (Chen et al., 2019). Insular function is not fully understood; apart from its role in sensorimotor processing (Uddin et al., 2017) and involvement in cognition (Seeley et al., 2007), the insula is also part of the limbic system and functions as its connection with the auditory system (Allen et al., 2008), as well as an intersection between the salience and default mode network (Kann et al., 2016). In general, a leftward lateralization in insular surface was reported (Jakab et al., 2012), due to the left hemisphere dominance in language gesture lateralization that could be even more pronounced in people using sign language (Allen et al., 2008). The reported right hemisphere dominancy of insula is in agreement with its involvement in the attentional orientation to salient stimulus, interoception, and physiological arousal (Kann et al., 2016), and similar findings were also reported by Watkins et al. (2001) and Chiarello et al. (2013).

Based on our previous results showing the minimal effects of hearing loss (different degree of presbycusis) on the gray and white matter of the auditory cortex and pathway (Profant et al., 2014) that were also confirmed in the current study (M2), and considering the strong influence of aging on the data that could potentially cover the tinnitus and hearing loss related changes, a separate model (M3) was tested for the effects of hearing loss (different degree of presbycusis) and tinnitus only on data from the elderly volunteers, to produce more age coherent subgroups. Another investigated factor was the left/right difference of reported variables as a proxy of brain asymmetry (laterality). Regarding the tinnitus, we additionally investigated the effects of its duration, severity and perception (sensation in one ear vs. both ears).

As previously described in our recent papers (Profant et al., 2013, 2014, 2015) and reviewed by Ouda et al. (2015), age related hearing loss *per se* has only a minimal effect on the chemical, functional, biochemical and morphometric parameters of the AC. Most of the findings, such as cortical thinning, decrease of the overall volume and surface area are induced by aging itself and are only minimally affected by the degree of hearing loss (Profant et al., 2014). Even deafness itself may not necessarily lead to morphometric changes in the AC (Penhune et al., 2003), however asymmetric hearing loss can cause cortical changes outside the AC (Li et al., 2013). Our data support the idea that symmetrical hearing loss has only a minimal effect on the morphometry of the AC. We found one small exception (with borderline significance) in the HG, caused by the presence of the expressed presbycusis in our data.

The observed decrease of the insular surface due to tinnitus, although side specificity was not tested, might also alter the previously reported leftward dominance. Insular involvement in tinnitus especially tinnitus distress was, along with the amygdala and anterior cingulate cortex, proposed in the wider network theory by De Ridder et al. (2014). The gating theory of tinnitus by Rauschecker et al. (2010) that focuses on limbic-corticostriatal-thalamic circuits also admits insular involvement, but not as a dominant structure in the persistence of tinnitus. Both of these theories are in line with our finding of increased volume in the amygdala and hippocampus, caused by the presence of tinnitus. The functional involvement of limbic structures in the tinnitus network was reported previously by Schmidt et al. (2013) and also Chen et al. (2017). One explanation of the potential tinnitus effect on auditory processing and vice versa is the bidirectional connection between the AC and limbic system (Weinberger, 2007; Munoz-Lopez et al., 2010), which is also supported by the reported enhanced functional connectivity between the temporal lobe and limbic system in tinnitus patients as reported by Chen et al. (2017). In contrast to the observed increase of the gray matter volume in our subjects with tinnitus, Landgrebe et al. (2009) reported a gray matter decrease in the left hippocampus in tinnitus subjects. The difference might be due to the different age and hearing thresholds of our tinnitus groups (although we have controlled for the age and hearing loss effects). We believe that our study using 3T MRI with dedicated FreeSurfer routines, which provides dedicated segmentation of the hippocampus and amygdala at individual levels, are more exact than the 1.5T MRI and voxel based morphometry analysis that were used in Landgrebe et al. (2009) study.

In this study, tinnitus also had a borderline effect on the AC, precisely on the PT. Our finding is in agreement with the decrease of the cortical thickness in the supratemporal gyrus (Aldhafeeri et al., 2012). An additional decrease of cortical thickness caused by tinnitus was also described in HG by other authors (Schneider et al., 2009; Allan et al., 2016), however we cannot confirm this finding. Some reports even describe an increase in the cortical thickness of the AC due to tinnitus (Husain et al., 2011; Boyen et al., 2013).

Although we did not observe tinnitus distress (examined by THI) related morphometric changes among our volunteers, such changes were reported by several previous studies specifically in the insula and bilaterally in superior and middle temporal cortices (Adjamian et al., 2014). Vanneste et al. (2015) reported a direct correlation between the tinnitus distress, loudness, duration, and its negative effect on the gray matter density (specifically in cerebellum and also to a lower extent in the primary AC). In general, tinnitus might lead to anxiety and depression that commonly cause morphometric changes within the limbic structures (Bora et al., 2012; Sandu et al., 2017). However, our tinnitus groups had very small variance of the tinnitus distress and also low results of THI, therefore they only suffered from very mild depression or anxiety due to tinnitus. Tinnitus distress also enhances connectivity between the temporal cortex and amygdala and hippocampus (Chen et al., 2017). Tinnitus laterality (unilateral vs. bilateral) and tinnitus duration showed no influence on any of the morphometric parameters within the explored sites, which is in agreement with literature (Adjamian et al., 2014; Yoo et al., 2016).

We are aware of the potential limitations of our study. Firstly, coherency of the subject subgroups is important. Although we have initially established a strict criteria and patients/volunteers were strictly selected, it is possible to be even more precise. However, the strictness of criteria dramatically decreases the number of subjects involved and therefore the statistical significance of data. Furthermore, even in cases when tinnitus is accompanied by physiological audiometric results, more detailed examination shows the presence of hearing pathology, therefore it is illusive to recruit subjects with tinnitus and normal hearing. Another factor is the limitation of the MRI examination and its precision. In contrast to human MRI studies, a histopathologic post-mortem examination of animal brain tissue shows morphometric changes due to hearing loss, and also the presence of tinnitus (Syka, 2002; Eggermont and Roberts, 2015; Ouda et al., 2015). Current up-to-date 3T systems, with dedicated multi-channel head coils, could produce images of better signal to noise ratio than the 3T Trio system with 12-channel head coil used in this study, and there is also the potential for further improvement with 7T MRI systems. However, it is questionable that this potential improvement in sensitivity would suffice to detect such subtle morphologic changes by MRI. A final complication of our data processing was the effect of multiple comparisons on the data. After a strict correction for multiple comparisons, previously significant data did not survive—specifically data related to tinnitus and hearing loss, therefore the term borderline significance was used for their description (in such cases for better clarity we provide both the corrected and uncorrected *p*-values).

## Conclusion

Our study revealed age related structural changes within the several cortical regions involved in the auditory processing and tinnitus network. Regarding the effect of tinnitus, our results show a trend specifically within the limbic structures. The structure of the auditory system is dominantly affected by aging and additionally we were also able to identify borderline hearing loss related changes.

A lack of significant changes caused by tinnitus and hearing loss in the auditory cortex can be attributed to several factors, such as the limitations of MRI resolution, signal to noise ratio, strictness of the inclusion criteria, number of participants. The strong prevalence of age effect shows that the coherence of subject subgroups is essential for the credible detection of tinnitus and hearing loss related changes in potential future studies.

## Data Availability Statement

The raw data supporting the conclusions of this article will be made available by the authors, without undue reservation.

## Ethics Statement

The studies involving human participants were reviewed and approved by Ethics Committee of the University Hospital Motol, in Prague. The patients/participants provided their written informed consent to participate in this study.

## Author Contributions

OP: manuscript preparation, examination, auditory examination, statistical analysis, and work load cca 25%. AŠ: MR examination, statistical analysis, manuscript preparation, and work load cca 20%. JT: MR examination, statistical analysis, and work load cca 15%. VS: auditory examination, database administration, and work load cca 13%. DK: auditory examination, database administration, and work load cca 12%. JSB: auditory examination, database administration, and work load cca 5%. JS: manuscript preparation, project overview, and work load 10%. All authors contributed to the article and approved the submitted version.

## Conflict of Interest

The authors declare that the research was conducted in the absence of any commercial or financial relationships that could be construed as a potential conflict of interest.

## Abbreviations

AC: auditory cortex
Amg: amygdala
O-HL-NT: aged group with expressed presbycusis
eTIV: estimated intracranial volume
HEAR: presbycusis presence/absence
HG: Heschl gyrus
HP: hippocampus
Ins: anterior insula
LAT: ROI laterality
O-NH-NT: aged group with mild presbycusis
PH: gyrus parahippocampus
PT: planum temporale
ROI: region of interest
SNHL: sensorineural hearing loss
O-HL-T: aged group with tinnitus and expressed presbycusis
TIN: tinnitus presence/absence
THI: Tinnitus Handicap Inventory questionnaire
O-NH-T: aged group with tinnitus and mild presbycusis
Y-NH-T: young group with normal hearing and tinnitus
V1: primary visual cortex
Y-NH-NT: young control group.
