# Supplementary material for: The Influence of Aging, Hearing, and Tinnitus on the Morphology of Cortical Gray Matter, Amygdala, and Hippocampus
Source: Front Aging Neurosci. 2020 Dec 4;12:553461. doi: 10.3389/fnagi.2020.553461 (PMC7746808; doi:10.3389/fnagi.2020.553461)
Supplement: Supplementary file 1 [file Table_1.DOCX]

List of specifications of particular models used in the paper. Each model contains the specification of the full model and the simplified version. Formula of the Model is constructed as followed: dependent variable ~ explanatory variables (fixed effects; connected by +), interaction of variables (connected by : symbol).

In Model formula, explanatory variables of interest are listed with capital letters. Covariates of no research interest (sex, etiv – estimated total intracranial volume, method – method for estimation of hippocampal/amygdalar volume) are listed with lowercase letters and their p-values were not evaluated for multiple comparison correction. P-values of variables of interest (both uncorrected p-values and p-values corrected by multiple comparisons by Holm method) are summarized in Table 1.

**M1 full**

thickness ~ LAT + REGION + AGE + sex + LAT:REGION + LAT:AGE + REGION:sex + REGION:AGE + LAT:REGION:AGE

**M1 simplified**

thickness ~ REGION +AGE + REGION:AGE

**M2 full**

thickness ~ TIN + LAT + AGE + sex

area ~ TIN + LAT + AGE + sex + etiv

**M2 simplified**

HG: thickness ~ AGE

area ~ LAT + AGE + etiv

PT: thickness ~ AGE

area ~ LAT + etiv

V1: thickness ~ AGE

area ~ LAT + etiv

PH: thickness ~ LAT + AGE + sex

area ~ LAT + etiv

Ins: thickness ~ AGE

area ~ TIN + LAT + etiv

**M3 full**

thickness ~ TIN + PRESB + LAT + AGE+sex

area ~ TIN + PRESB + LAT + AGE+sex + etiv

**M3 simplified**

HG: thickness ~ AGE

area ~ PRESB + LAT + etiv

PT: thickness ~ AGE

area ~ TIN + LAT + AGE + sex + etiv

V1: thickness ~ LAT + AGE

area ~ LAT + etiv

PH: thickness ~ LAT + AGE + sex

area ~ LAT + AGE + etiv

Ins: thickness ~ AGE

area ~ etiv

**M4 full**

thickness ~ TIN_LATERALITY + LAT + AGE + sex + TIN_LATERALITY:LAT

area ~ TIN_LATERALITY + LAT + AGE + sex + etiv + TIN_LATERALITY:LAT

**M4 simplified**

HG: thickness ~ AGE

area ~ 1 (null model)

PT: thickness ~ AGE

area ~ AGE

V1: thickness ~ AGE

area ~ AGE

PH: thickness ~ AGE

area ~ AGE

Ins: thickness ~ 1 (null model)

area ~ AGE

**M5 full**

volume ~ TIN + PRESB + LAT + AGE + sex + etiv+method

**M5 simplified**

Hippocampus: volume ~ TIN + LAT + AGE + etiv + method

Amygdala: volume ~ TIN + LAT + AGE + etiv + method

**M6 full**

a) volume / area ~ TIN_LENGTH + LAT + AGE + sex + etiv

b) volume / area ~ THI + LAT + AGE + sex + etiv

**M6 simplified**

PT: a) area ~ LAT + AGE

b) area ~ LAT + AGE

Hippocampus: a) volume ~ LAT + etiv

b) volume ~ LAT + etiv

Amygdala: a) volume ~ LAT + etiv

b) volume ~ LAT + etiv
